# Structure-guided design of *Serratia marcescens* short-chain dehydrogenase/reductase for stereoselective synthesis of (*R*)-phenylephrine

**DOI:** 10.1038/s41598-018-19235-y

**Published:** 2018-02-02

**Authors:** Jai-Shin Liu, Yi-Chia Kuan, Yu Tsou, Tung-Yueh Lin, Wen-Hwei Hsu, Ming-Te Yang, Jong-Yih Lin, Wen-Ching Wang

**Affiliations:** 1 Institute of Molecular and Cellular Biology & Department of Life Science, National Tsing Hua University, Hsinchu, 300 Taiwan; 2 Institute of Molecular Biology, National Chung Hsing University, Taichung, 402 Taiwan; 3 Department of Mechanical Engineering, National Chung Hsing University, Taichung, 402 Taiwan

## Abstract

Bioconversion is useful to produce optically pure enantiomers in the pharmaceutical industry, thereby avoiding problems with side reactions during organic synthesis processes. A short-chain dehydrogenase/reductase from *Serratia marcescens* BCRC 10948 (*Sm*SDR) can stereoselectively convert 1-(3-hydroxyphenyl)-2-(methylamino) ethanone (HPMAE) into (*R*)-phenylephrine [(*R*)-PE], which is marketed medically as a nasal decongestant agent. The whole-cell conversion process for the synthesis of (*R*)-PE using *Sm*SDR was reported to have an unexpectedly low conversion rate. We reported the crystal structure of the *Sm*SDR and designed profitable variants to improve the enzymatic activity by structure-guided approach. Several important residues in the structure were observed to form hydrophobic clusters that stabilize the mobile loops surrounding the pocket. Of these, Phe98 and Phe202 face toward each other and connect the upper curvature from the two arms (i.e., the α7 helix and loopβ4–α4). The mutant structure of the double substitutions (F98YF202Y) exhibited a hydrogen bond between the curvatures that stabilizes the flexible arms. Site-directed mutagenesis characterization revealed that the mutations (F98Y, F98YF202Y, and F98YF202L) of the flexible loops that stabilize the region exhibited a higher transformation activity toward HPMAE. Together, our results suggest a robust structure-guided approach that can be used to generate a valuable engineered variant for pharmaceutical applications.

## Introduction

Bioconversion approaches to producing optically pure enantiomers are pursed in the pharmaceutical industry because such approaches reduce environmental impacts while sustaining high selectivity, specificity, and mild reaction conditions^1–4^, as well as avoiding side-reaction problems during the organic-synthesis process^5^. Short-chain dehydrogenses/reductases (SDRs) that exhibit an asymmetric reduction of a wide range of ketones to the corresponding chiral alcohols are valuable stereoselective biocatalysts^6^. They are clustered into five subfamilies according to three motif segments consisting of 40 conserved residues, covering the coenzyme-binding and active site regions^7^. Of these conserved regions, seven structural elements are used to categorize the classical and extended SDR subfamilies based on conserved sequence motifs^8^. Despite having heterogeneous sequences, the available structures of SDRs demonstrate a typical *Rossmann*-fold scaffold containing a NADP binding site at N-terminal region and a variable loop at the C-terminus, thus enabling the recognition of a wide variety of substrates^7,9^. Because they provide diverse substrate pockets, SDRs have great potential for the biocatalytic conversion of chemicals, blood detection, and the production of pharmaceutical intermediates^10–14^.

(*R*)-Phenylephrine [(*R*)-PE], a potent sympathomimetic drug, is used clinically as a systemic medicine to dilate the pupils and increase blood pressure, and it is widely used as an over-the-counter nasal decongestant^15^. Industrial chemical synthesis of (*R*)-PE yields a mixture of (*R*)- and (*S*)-form enantiomers. Thus, an asymmetric hydrogenation method is required for producing optically pure (*R*)-PE^16^. A few schemes for the asymmetric synthesis of (*R*)-PE have been developed^17–20^. However, such chemical processes demand the use of high pressure and temperature, as well as environmentally damaging organic solvents^21^.

We have previously identified an SDR from *Serratia marcescens* BCRC 10948 (*Sm*SDR) that was able to convert 1-(3-hydroxyphenyl)-2-(methylamino) ethanone (HPMAE) into (*R*)-PE. *Sm*SDR is a 3-oxoacyl-acyl-carrier-protein (OACP) reductase of the classical SDR subfamily that catalyzes the reduction of its authentic substrate, OACP, to (3 *R*)-3-hydroxy-ACP, participating in the fourth step of 16-carbon palmate biosynthesis^22,23^. Hence, expectedly, the whole-cell conversion process to synthesize (*R*)-PE using *Sm*SDR has a relatively low conversion rate^21,24,25^. To engineer *Sm*SDR with higher activity and improve this biotransformation process, a detailed structure-function analysis of *Sm*SDR and *Sm*SDR-substrate interactions is necessary. In addition, the SDR proteins have been used to alter coenzyme and substrate specificity for biological application^26,27^.

We report the crystal structure of *Sm*SDR in its apo and NADPH-liganded forms. To obtain the requisite industrial properties, we further utilized the structure-guided approach to engineer mutants, enhancing their activity by stabilizing the mobile loops around the pocket. These structurally based engineered mutants exhibited raised conversion yields and productivity, suggesting a valuable engineered variant for pharmaceutical applications.

## Results

### Structures of the apo-form *Sm*SDR, F98AF202L *Sm*SDR, F98LF202L·NADPH, and F98YF202Y variants

**Figure 1:**
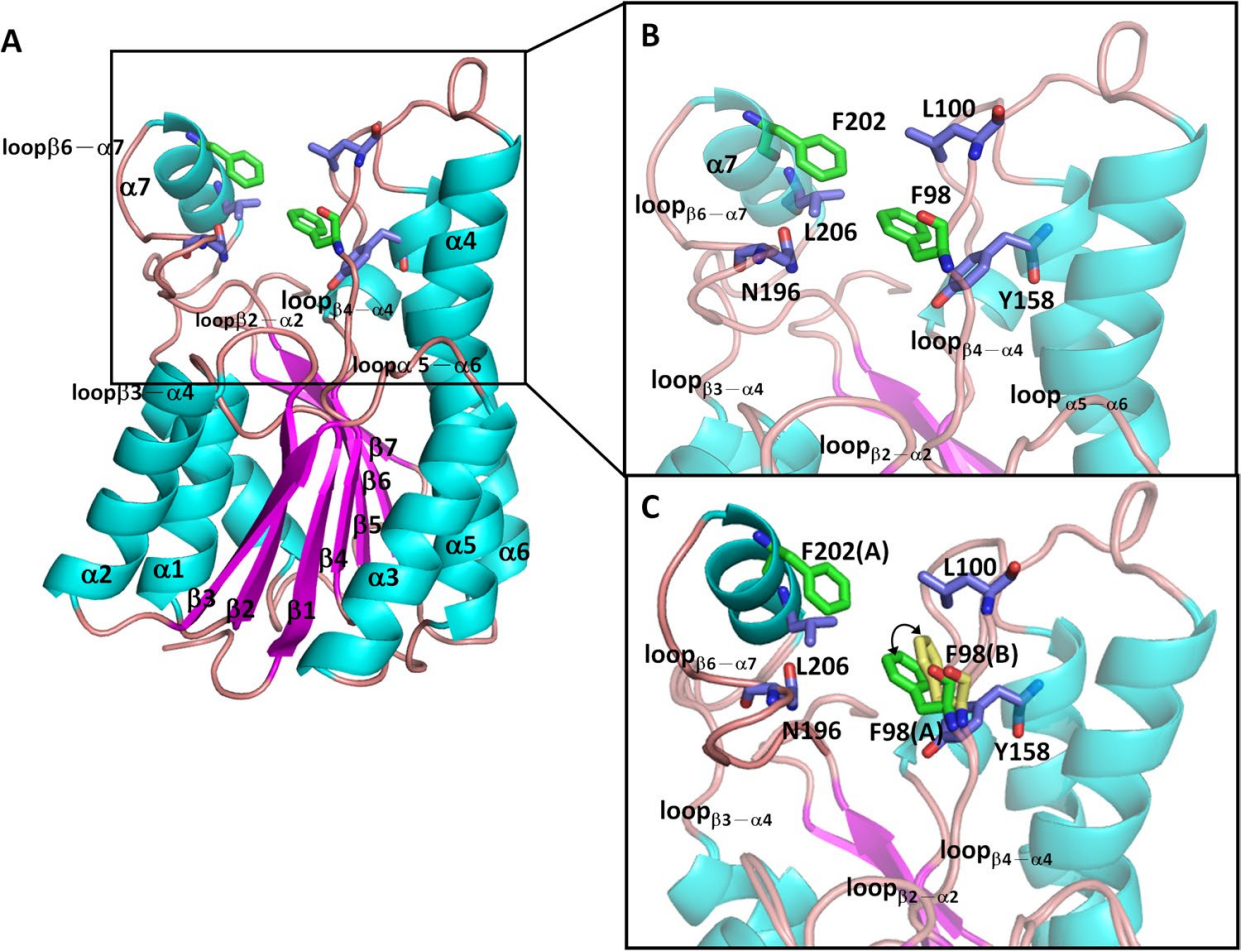
Structure of *Sm*SDR. (**A**) Structure of the apo form of *Sm*SDR. A presumed NADPH-binding cleft is surrounded by β2–α2, β4–α4, β3–α4, α5–α6, β6–α7, and an α7 helix. The α-helix, β-sheet, and loop are colored cyan, magenta, and brown, respectively. Phe98 and Phe202 are depicted as green stick models. The residues of the hydrophobic cluster are colored as purple stick models. The carbon, nitrogen, and oxygen atoms are shown as green, blue, and red, respectively. (**B**) Close-up view of the hydrophobic core on the presumed binding pocket. Phe98 (green) from the β4–α4 loop and Phe202 (green) from the α7 helix face toward each other and contact with the L100, Y158, N196, and I206 residues (purple). (**C**) Superposition of subunit A and B reveals flexible side chain of the Phe98.

The structure of the apo form of *Sm*SDR was solved to 1.47 Å (*Sm*SDR), revealing a homo-dimer (AB) in the crystal. Weak or negative density was seen at two segments of subunit B (residues 40–50 and 191–208), indicating a flexible region. Subunit A comprises residues 5–249, whereas subunit B comprises residues 2–40, 51–190, and 209–377. Subunit A contacts with subunit B through α1, α6 helices, loop_α8–β7_, and a region after the β7 sheet (Fig. 1A and Fig. S1). A tight homotetramer is created with two homodimers related by two-fold crystallographic symmetry (Fig. S2A). Furthermore, size-exclusion chromatography of the purified *Sm*SDR demonstrated a single peak that corresponds to the size of a tetramer (Fig. S2B), confirming that *Sm*SDR is assembled into a tetramer, in accord with the tetrameric structure seen for homologues^28^.

Each subunit is intertwined to form an α/β/α-fold barrel, of which the seven-β-stranded central sheet is flanked by six α-helices on either side, showing a nucleotide-binding *Rossmann* scaffold. The presumed active-site pocket is surrounded by five loops (β2–α2, β3–α4, β4–α4, α5–α6, and β6–α7) and an α7 helix (acting as a lid) (Fig. 1A).

Two phenylalanines, Phe98 from the β4–α4 loop (residues 94–107) and Phe202 from the α7 helix, face toward each other, thus constituting a bridge structure over the top of the binding pocket (Fig. 1A and B). Notably, they contact with L100, Y158, N196, and I206 to form a hydrophobic cluster on the peripheral region of the pocket. Superposition of subunit A and subunit B of apo-*Sm*SDR reveals a slight shift near Phe98 at the β4–α4 loop. Of note, there is 1.85-Å deviation between the phenyl Cζ atoms of Phe98 [Cζ(Phe98:A)-Cζ (Phe98:B)] of superimposed subunits, suggesting that Phe98 resides at a flexible region (Fig. 1C).

### The active site of *Sm*SDR and HPMAE complex model

**Figure 2:**
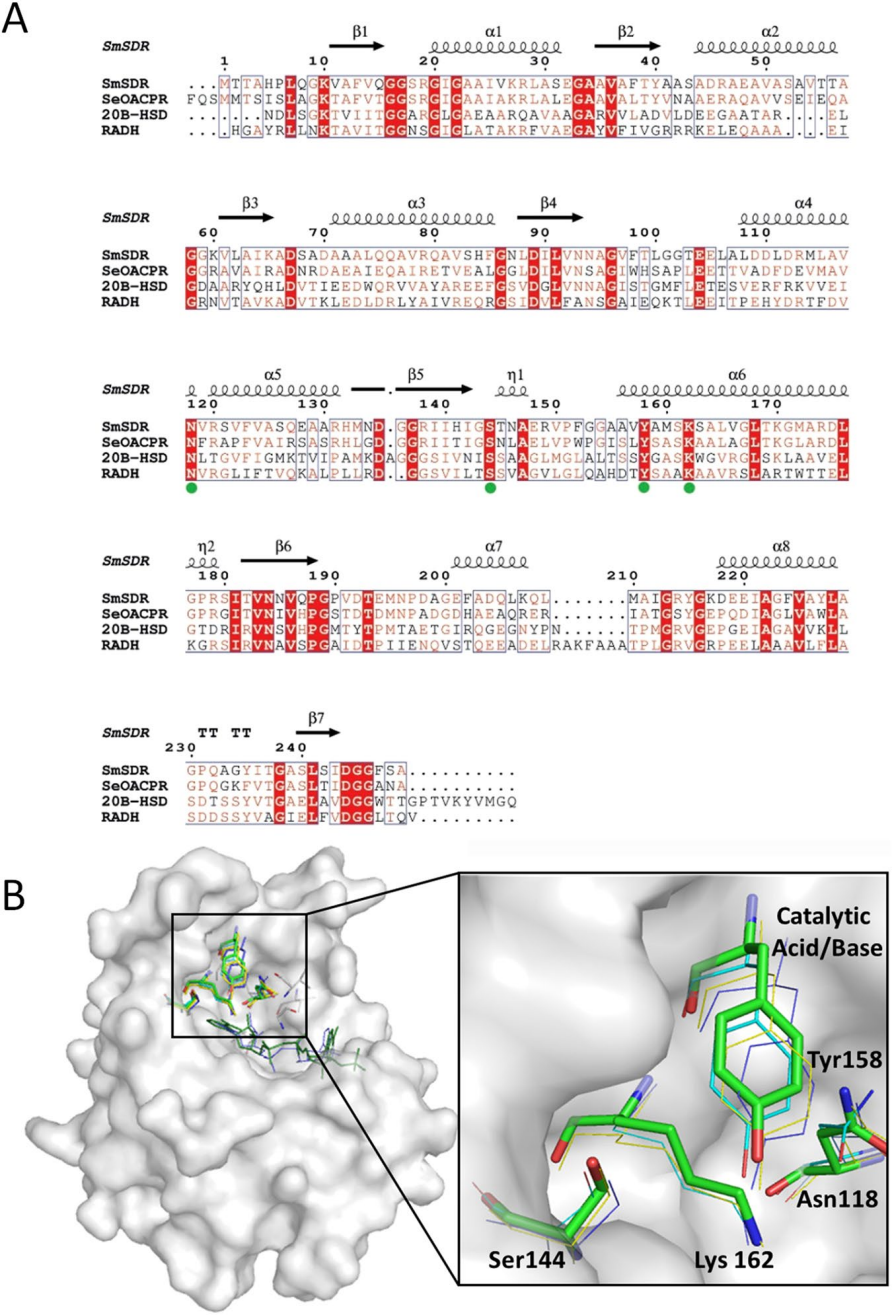
Structure-based alignment of the homologous structures of SDRs. Secondary structural elements are presented above the sequence. The β-strands (β1–7) and α-helices (α1–8) are numbered from the N terminus. TT, β-turns; η (η1, η2), 3_10_ helix. The conserved catalytic tetrad residues are indicated as green circles. (**B**) Close-up view of the superimposed conserved catalytic tetrad. The tetrad residues in *Sm*SDR are drawn as thick stick model.

Structure-based alignment of the homologous structures of SDRs reveals that *Sm*SDR consists of a conserved tetrad (Asn-Ser-Tyr-Lys) present in most SDR enzymes (Fig. 2A): Asn118, Ser144, Tyr158 and Lys162^29,30^. Asn118 makes a strong hydrogen bond with Ser161, which connects to two nearby catalytic residues Tyr158 and Lys162 through H bonds, forming an extensive hydrogen network. Asn118 also contacts with Gly96, Val97, Val117, Val119, Val122, Ser161, and Leu165, analogous to those of Asn111 in alcohol dehydrogenase^31^. Superposition among *Sm*SDR, *Sinorhizobium meliloti 1021* 3-oxoacyl-[ACP] reductase (*Sm*OACPR; pdb code: 3V2G), *Comamonas testosteroni* 3α/17β-hydroxysteroid dehydrogenase (pdb code: 2HSD)^32^ and *Ralstonia sp*. alcohol dehydrogenase (pdb code: 4I5E)^30^, suggesting a common catalytic mechanism shared by these dehydrogenases (Fig. 2A and B)^30,31^.

**Figure 3:**
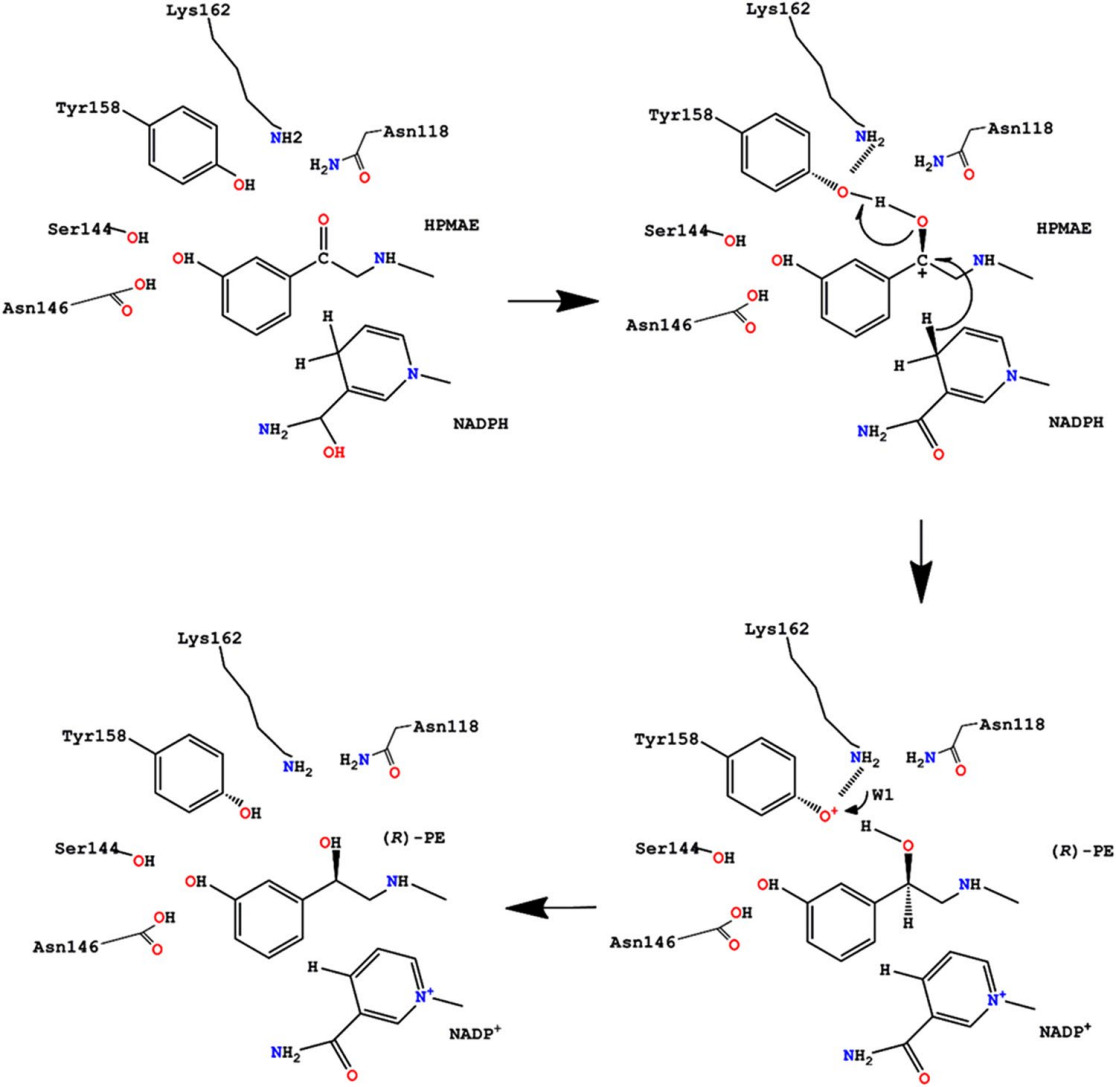
Proposed catalytic mechanism of *Sm*SDR for the conversion of HPMAE into (*R*)-PE. The hydrogen atom of the nicotinamide approaches the C3 atom of HPMAE, initiating a hydride transfer to the atom. Subsequent protonation of Tyr158 by a water molecule then reduces the carbonyl group to ethanol.

We therefore prepared the *Sm*SDR-NADPH-HPMAE complex model using an *in silico* docking method^33,34^. The top-ranking docked pose of HPMAE shows HPMAE-interacting environment (Fig. S3): (1) The C4 atom of the nicotinamide ring from NADPH contacts with the C3 atom of HPMAE; (2) the carbonyl O atom interacts with the catalytic residue Tyr158; (3) the aromatic ring of HPMAE aligns with that of Phe98 to some extent, yielding displaced π stacking force; and (4) the hydroxyl group of the HPMAE contacts with Ser144 and Asn146. On the basis of this model, we propose a catalytic mechanism that converts HPMAE into (*R*)-PE through *Sm*SDR at the expense of NADPH to yield NADP^+^ (Fig. 3): Tyr158 acts as a key catalytic player, the nearby Lys162 interacts with the hydroxyl of the tyrosine that lowers the p*Ka*, Ser144 stabilizes the substrate, and Asn118 maintains the active-site framework to build up a proton relay environment as suggested by Filling *et al*.^31^.

### Structure-guided mutagenesis on Phe98 and Phe202

**Figure 4:**
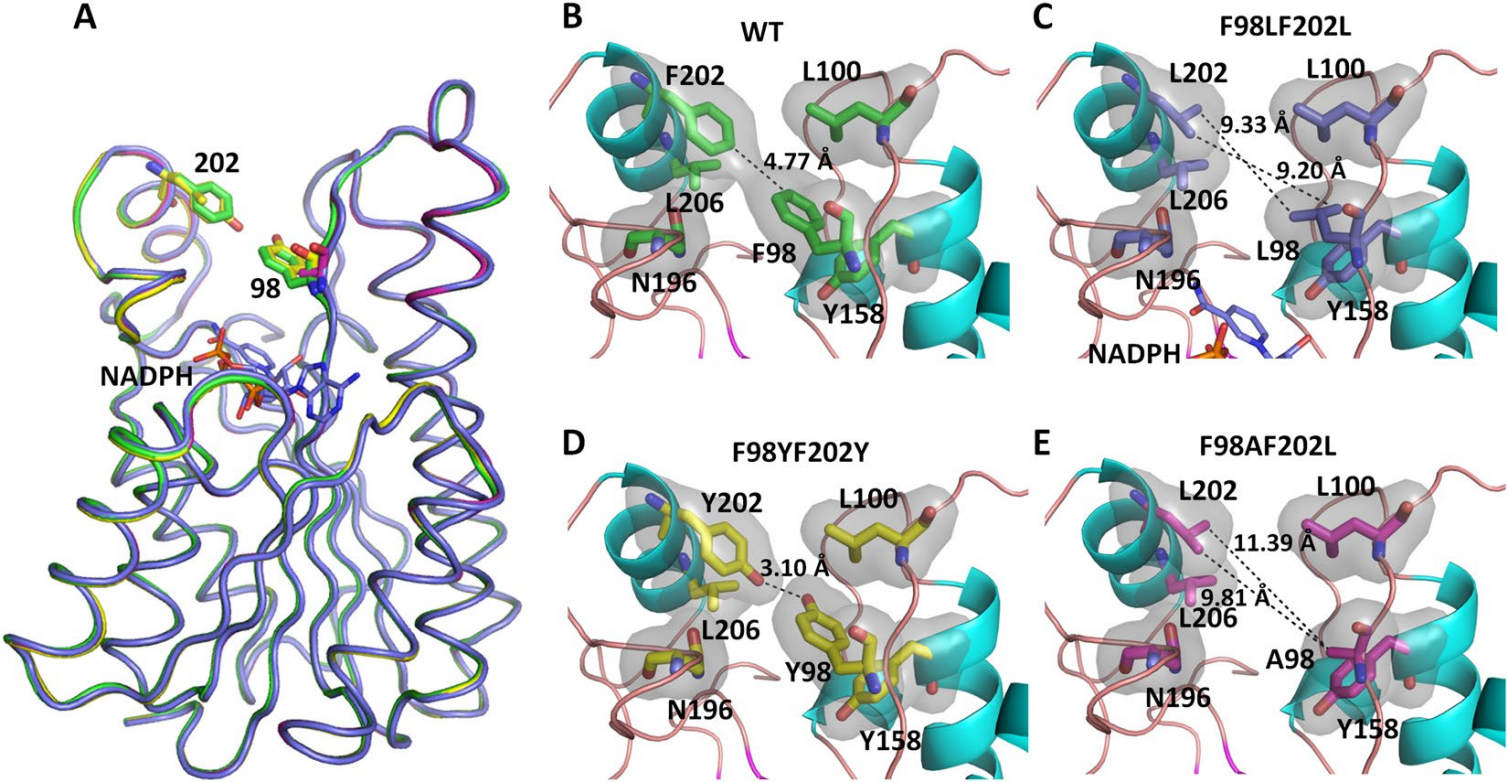
Comparison of the hydrophobic surface in the F98-F202 region. (**A**) Superposition of four *Sm*SDRs [apo form of *Sm*SDR (green), F98L·F202L·NADPH (purple), F98Y·F202Y *Sm*SDR (yellow), and F98A·F202L (magenta),]. NADPH is depicted as a stick model, and the nitrogen, oxygen, and phosphorus atoms are depicted as purple, red, and orange, respectively. (**B**) Apo form of *Sm*SDR, (**C**) F98L·F202L·NADPH·*Sm*SDR, (**D**) F98Y·F202Y·*Sm*SDR, and (**E**) F98A·F202L·*Sm*SDR. The hydrophobic surface was generated according to the Kyte‐Doolittle hydrophobic scale^65^ and is colored gray. The α-helices and loops are colored cyan and orange. The residues are depicted as thick stick models. Dashed lines indicate the distance between the depicted atoms.

Given that Phe98 and Phe202 are situated at a relatively hydrophobic, flexible region over the top of the binding pocket, we sought to evaluate whether engineering of these sites affected enzymatic activity. We have therefore generated and expressed point-mutation variants using the *Escherichia coli* expression system: F98Y, F98YF202L and F98YF202Y. We were able to obtain good-diffracting crystals of F98AF202L, F98YF202Y, as well as F98LF202L that grew in the condition consisting of NADPH. Given an atomic resolution diffraction of apo-form (1.47 Å, Fig. S4A), the structure of the mutant F98AF202L was determined to 1.87 Å, showing a clear electron density map of F98A and F202L (Fig. S4B). The structure of the double-tyrosine-substituted mutant F98YF202Y was solved to 1.47 Å, revealing a clear electron density map of the mutated tyrosines (Fig. S4C). In addition, we obtained the F98LF202L·NADPH complex crystal using the soaking method. The 1.90-Å *Sm*SDR mutant structure (F98LF202L·NADPH) was determined, showing a clear density map of the mutated residues F98L and F202L (Fig. S4D). Structural comparison of the mutant structures (F98AF202L, F98LF202L, and F98YF202Y) demonstrated an overall homologous fold (Fig. 4A). A close-up view of the region between residues 98 and 202 showed that there is significant conformational alteration: (1) no contacts between residues 98 and 202 in F98AF202L [Cβ(Ala98)–Cδ1(Leu202): 9.81 Å; Cβ(Ala98)−Cδ2(Leu202): 11.39 Å] and in F98LF202L [Cδ1(Leu98)–Cδ2(Leu202): 9.20 Å and Cδ2(Leu98)–Cδ1(Leu202): 9.33 Å]; and (2) an additional H bond between the hydroxyl groups of Y98 and Y202 in F98YF202Y [hydroxyl O(Tyr98)–O(Tyr202): 3.10 Å] (Fig. 4B–E). Thus, the replacement of the bulky aromatic chain with a shorter side chain at the region of residue 98 greatly led to the loss of the hydrophobic contacts between two arms. Conversely, F98YF202Y that introduces an additional hydroxyl group on the phenyl side chain creates a strong H bond.

In the F98LF202L·NADPH structure, a clear density map near the bottom of the pocket in Subunit A was observed, which could be modeled as NADPH. Superposition of the apo form of *Sm*SDR, F98AF202L, F98LF202L·NADPH, and F98YF202Y·*Sm*SDR demonstrated an essentially identical fold. Structural comparison indicated relatively low RMSD values among these structures [apo vs. F98AF202L: 0.11 Å (223 Cα atoms); apo vs. F98YF202Y: 0.14 Å (234 Cα atoms); and apo vs. F98LF202L·NADPH: 0.14 Å (226 Cα atoms)] (Fig. 4A). Unlike the conformational change altered in the β-ketoacyl-ACP reductase from *E*. *coli*^35^, *Sm*SDR reveals hardly any conformational change upon NADPH binding, providing a stable configuration of the cofactor-binding-site framework for catalysis.

**Figure 5:**
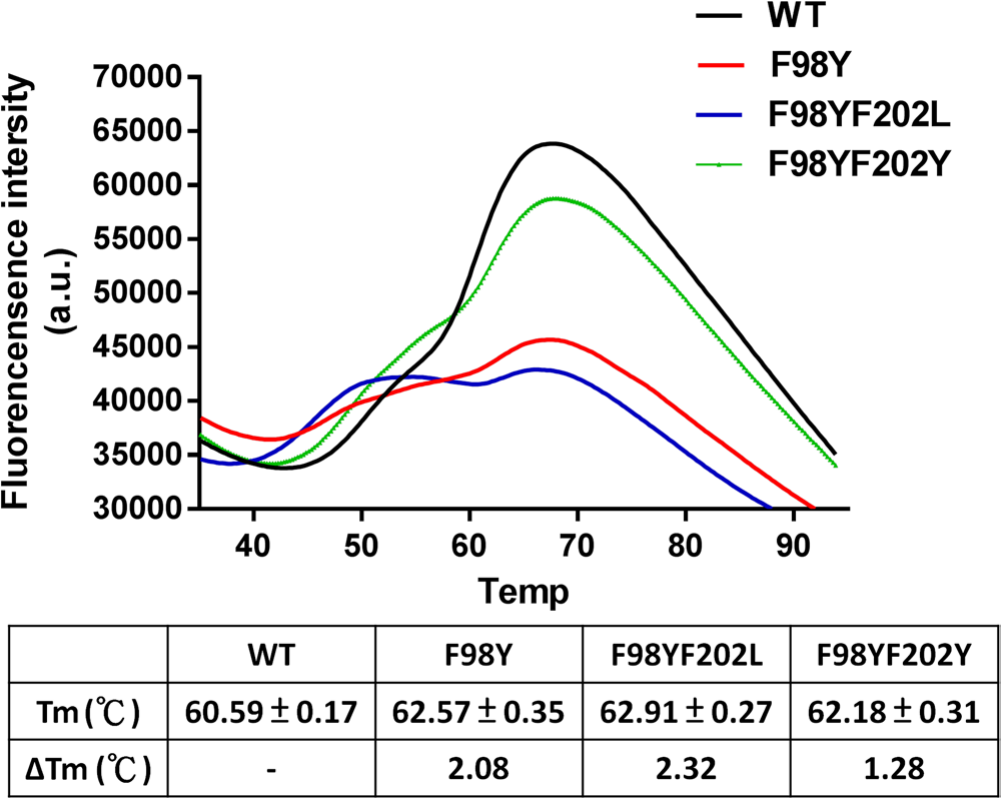
Differential scanning fluorimetry (DSF) analysis of *Sm*SDR variants. Tm is denoted as the midpoint of the unfolding transition of each protein. Tm and ΔTm values per variant are shown in the bottom table.

We next evaluated the mobility of these variants based on a thermal stability shift assay that provides a quantitative measure of the enzymatic thermostability^36^. As compared with the wild-type enzyme, F98Y and F98YF202Y had an increased thermal shift (2.08 and 1.28 °C) (Fig. 5). F98YF202L mutant exhibited a more flat peak with biphasic melting temperatures (45.59 and 62.91 °C), implicating a two-state denaturation process for F98YF202L. These results suggested that the introduction of a hydroxyl moiety on the aromatic side chain at residues 98 could enhance the thermostability.

WT, F98Y, F98YF202L and F98YF202Y were subjected for enzymatic kinetic analysis. As shown in Table 1, there was a lower *K*_*m*_ value for F98Y, F98YF202L and F98YF202Y, indicating an increased binding affinity with substrate. A slightly lower *k*_*cat*_ value was derived for each variant, which led to a comparable *k*_*cat*_/*K*_*m*_ value (Table 1). We next evaluated the transformation yield of PE from HPAME using *E*. *coli*-expressing variants. Notably, F98Y, F98YF202L and F98YF202Y all exhibited a higher bioconversion yield than did the wild-type enzyme. Of those, F98YF202L had the highest transformation activity as compared with wild-type (1.57-fold). These results suggest that F98YF202L with a biphasic feature maintains its flexibility and stability, hence higher transformation power.

**Table 1:** Kinetics parameters for *Sm*SDR variants.

	Wild-type	F98Y	F98YF202L	F98YF202Y	A42S
*K*_*m*_ (NADPH)	139.70 ± 21.82 μM	116.90 ± 22.86 μM	114.00 ± 17.46 μM	91.41 ± 10.89 μM	101.10 ± 39.53 μM
*k*_*cat*_ (S^−1^)	7.69 ± 0.55	7.04 ± 0.57	7.40 ± 0.46	6.18 ± 0.76	5.00 ± 0.76
*k*_*cat*_ */K*_*m*_	0.06	0.06	0.06	0.07	0.05
PE yield (mM)^*^	2.13 ± 0.03	3.05 ± 0.06	3.35 ± 0.03	2.84 ± 0.01	2.86 ± 0.10

*The PE yield was normalized based on the quantity of the expressed *Sm*SDR in *E*.*coli*-expressing cells.

### Comparison with NADPH-preferring SDR structures

We compared SDR structures that prefer NADPH as a cofactor involved in the hydride transfer of NADPH^37^. On the basis of Dali analysis^38^, with the F98LF202L·NADPH structure serving as the query, we searched for similar structures with high sequence identity. Of those having a high Z score (>10), three enzymes were observed to share high sequence identity (>40%) with *Sm*SDR: *Sinorhizobium meliloti 1021* 3-oxoacyl-[ACP] reductase (*Sm*OACPR; 57% identity; pdb code: 3V2G)^39^, *Gluconobacter oxydan* putative reductase (*Go*POR; 43% identity; pdb code: 3WTB)^40^, and *Synechococcus elongatus PCC 7942* OACPR, (*Se*OACPR; 42% identity; pdb code: 4DMM) (Fig. S5)^41^. Of these, only *Se*OACPR that behaves as an NADPH-preferring enzyme has an available NADPH-liganded structure. Superposition of these structures revealed an overall homologous architecture (r.m.s: 0.68–0.99 Å for the Cα backbone; Fig. S5A) except for loop_β6–α7,_ of which the contacts are conserved. Alignment of the binding pockets of *Se*OACPR and *Sm*SDR showed a number of residues contacting with NADPH (<3.8 Ǻ) at *Sm*SDR: Gly16, Ser18, Arg19, Ile21, Ala41, Ala42, Asp67, Asn94, Asn128, Tyr158, Lys162, Gly189, Val191, and T193 (Fig. S5B). Two additional contacts with the 2′-phosphate group of the nicotinamide-ribose structure are observed in the loop_β2–α2_ of the *Se*OACPR·NADPH owing to the heterogeneity of this loop (Ala42Ser43 in *Se*OACPR as compared with Ala41Ala42 in *Sm*SDR): (i) Ser41(Oγ)–phosphate(O3), 3.37 Å; and (ii) Ser42(N)–phosphate(O2), 2.82 Å. The other crucial H-bond with the 2′-phosphate group of the nicotinamide ribose structure is from the conserved basic arginine located at the end of the glycine motif (Ser18Arg19 in *Sm*SDR; Ser17Arg18 in *Se*OACPR) (Fig. S5B). Thus, *Sm*SDR and *Se*OACPR belong to a subgroup (cP1) of classical SDRs that acquire a conserved basic residue (Lys/Arg) to interact with NADPH^8^. The 2′-phosphate acts not only as the preference of the coenzyme but also stabilizes the loop_β2−α2_.

We also evaluated the effect of A42S, which was previously assumed to introduce an additional contact

between the 2′-phosphate group of NADPH involving the loops_β2–α2_ (Fig. S5B), mimicking that in *Se*OACPR^42^. A42S had a comparable *k*_*cat*_/*K*_*m*_ value as compared with the wild-type enzyme. A higher PE conversion yield was obtained in the A42S variant as compared with wild-type (1.34-fold increase) (Table 1), indicating a slightly beneficial effect in biotransformation. Thus, engineering of these sites (residues 98 and 202) at the α7 helix and loop β4–α4 to enhance the stability, as well as of those residues assumed to interact with the 2′-phosphate group of NADPH to augment the contacts with NADPH^30^, offers a useful strategy for improving the conversion rate of HPMAE.

## Discussion

Of the cP1-type classical SDRs, *Sm*SDR and *Se*OACPR are two members that catalyze the stereoselective reduction of a ketone to its corresponding chiral alcohol^37,43^. Both *Sm*SDR and *Se*OACPR structures comprise a strictly conserved Asn-Ser-Tyr-Lys tetrad and a highly conserved Rossmann-fold N-terminal lobe that binds to the NADH/NADPH cofactor^10,14,44^. Based on the *Sm*SDRs’ structures analyses, the most sensitive sites of *Sm*SDR to temperature are identified on the surface region (α4, α7 and loop_β4–α4_ at the C-terminal segment; Fig. S6A–D) of the C-terminal lobe. Above all, the α7 helix and loop_β4–α4_ with high B-factor values extends from the compact N-lobe Rossmann domain, suggesting a flexible region to accommodate various substrates.

Targeted mutagenesis guided by structural analysis to engineer enzymes of acquired features has been emerged as a useful strategy^45^. F98 and F202 that protrudes from two arms contribute to hydrophobic contacts thus provide a potential site for engineering to enhance the enzymatic thermostability (Fig. 4) since they are less likely to disturb the catalytic framework and increase the likelihood for obtaining active variants with increased thermostability. Thermostability analysis revealed that the F98Y mutation with an additional hydroxyl group could effectively enhance the thermostability (∆Tm: 2.08 °C), whereas the F98YF202L showed a biphasic melting pattern (Tm: 45.59 and 62.91 °C). These variants exhibited relatively comparable *k*_*cat*_/*K*_*m*_ values, suggesting that the overall active-site framework is reserved. Interestingly, the whole-cell transformation showed that all three variants had a higher PE biotransformation yield, in which F98YF202L had the best performance. From the structural point of view, the phenyl moiety of Phe98 contacts with the aromatic ring of Tyr158 (Phe98(Cδ1)–Phe158 (Cγ), 3.70 Å), yielding an edge-to-face aryl-aryl interaction importantly to orient the precise position of the catalytic Tyr158^46^. F98Y is likely to introduce an additional H contact between the O atom of Tyr98 and N atom of Asn146, hence increasing the stability based on the F98Y structure model. The F98YF202Y mutation that introduces an extra H bond seems to strengthen the conformation rigidity, hence resulting in an increased HPMAE affinity. Yet, a lower *k*_*cat*_ value of F98YF202Y implicated that this mutation might restrict subsequent catalytic events possibly due to its inflexibility. On the other hand, F98YF202L that gains the interaction with Asn146 at residue 98 while introduces more flexibility at residue 202 as shown by its biphasic melting feature had the highest transformation yield. These results together suggest that engineering the thermally sensitive loops to strengthen protein stability while maintain its precise catalytic architecture and flexibility represents a robust structure-guided approach for pharmaceutical applications and that F98LF202L and F98YF202Y are profitable mutants^47^.

Engineering the cofactor affinity is an additionally useful strategy to improve the production efficiency^48,49^. From the practical point of view, NADH is a much economical cofactor (15 times price lower than NADPH) for production of (R)-PE by a cell-free method. It is even more desirable by the use of a whole-cell biocatalysis transformation process that directly produces NADH/NADPH using the media carbon source; log-phase *E*. *coli* culture generates three times higher concentrations of NADH than NADPH^48–50^. Analysis of the NADPH-liganded structures between *Sm*SDR and *Se*OACPR shows that the 2′-phosphate group of the nicotinamide-ribose points toward loop_β2–α2_. Substitution of alanine by serine at residue 42 (*Sm*SDR A42S) on this loop slightly enhanced biotransformation activity, despite comparable enzymatic activity. This might have implication that this mutation had a switch in cofactor specificity reported by Huang *et al*. and Lerchner *et al*.^30,51^. Further investigation would be needed to derive an NADH-preferring variant by targeting loop_β2–α2_ to increase whole-cell biocatalysis efficiency. In support of this notion, a complete switch of the cofactor specificity (from NADP^+^ to NAD^+^) has been reported by engineering this loop in *Ralstonia* sp. alcohol dehydrogenase, realizing a complete change in NADH/NADPH specificity (a factor of ~3.6 million)^30^. This also sheds lights into the future engineering of *Sm*SDR.

In summary, the apo-form and NADPH-liganded *Sm*SDR structures determined in this investigation reveal an unexpectedly mobile region near the entrance of the substrate-binding region. Together with the simulated HPMAE complex model, the catalytic mechanism of (*R*)-PE conversion is proposed. Rationally designed mutants of the mobile loops significantly improved the synthesis of (*R*)-PE. These results together suggest that stabilization of this flexible region (i.e. α7 helix and loop β4–α4) provides a robust means to engineer *Sm*SDR with higher transformation activity and a valuable variant F98YF202L.

## Materials and Methods

### **Cloning**, **expression and purification of*Sm*SDRs**

The *S*. *marcescens sdr* gene encoding *Sm*SDR protein was amplified by PCR from *S*. *marcescens* BCRC 10948 genomic DNA (GenBank: CP003959.1) using gene specific primers (Table S1). Chromosomal *S*. *marcescens* DNA was isolated as described previously^21^ and used as the template. The amplified product was inserted into the pET30a vector (Novagen, Inc., USA) to generate pET30a·*smsdr* plasmid, followed by transformation using *E*. *coli* BL21 (DE3) strain. The cells were cultivated in Luria-Bertani medium at 37 °C for 16 h until the OD_600_ reached 0.8. The overexpression *Sm*SDR protein was induced at 28 °C for 16 h by the addition of isopropyl-β-D-thiogalactopyranoside (IPTG) to a final concentration of 0.5 mM. The cells were collected by centrifugation at 6,000 × g at 4 °C for 15 min and resuspended in 30 ml of 50 mM sodium phosphate buffer (pH 7.0) containing 300 mM NaCl and followed by sonication. The cell debris was pelleted at 10,000 × g at 4 °C for 20 minutes and the resulting supernatant was loaded into a nickel affinity column (Novagen, Madison, WI, USA). The His6-tagged proteins were eluted using the buffer containing 200 mM imidazole. The purified protein was verified by SDS-PAGE analysis. Protein concentration was assayed by the Bradford method using bovine serum albumin as the standard^52^.

### Site-directed Mutagenesis

Mutated SDR genes were amplified by overlap extension PCR amplification^53^ using the site-directed mutagenesis primers listed in Table S1. The pET30a-*Smsdr* plasmid was used as the template. Each amplified gene was cloned into the *KpnI/SalI* site in pET30a vector and the *sdr*-inserted plasmid carrying requisite point mutation was individually introduced into *E*. *coli* BL21 (*DE3*). The mutated plasmid was confirmed by DNA sequencing.

### Crystallization and determination of the structure of *Sm*SDR

Crystallized *Sm*SDR experiments were performed by sitting drop method. Initial crystallization screening was used Oryx8 protein crystallization robot platform (Douglas Instruments, UK) and Crystal Screen Index, I, and II kits (Hampton Research), Clear Strategy Screen I and II kits (Molecular Dimension), Wizard kit (Emerald), and JB Screen classic HTS I and II kits (Jena Bioscience).

Optimized crystals reached to a maximum size under 20 °C within 14 days under the condition containing 50 mM HEPES buffer (pH 7.0), 40% (v/v) tacsimate (pH 7.0), 2 mM spermine, and 2 mM hexamine cobalt (III) chloride mixing equally with the protein solution (10 mg ml^−1^). NADPH-bound *Sm*SDR crystals were obtained by soaking with 100 mM NADPH made up in 50 mM HEPES buffer and then incubated at room temperature for 30 minutes.

Diffractions were collected at the BL15A1 using Rayonix MX300HE CCD area detector, National Synchrotron Radiation Research Center (NSRRC), Taiwan. All datasets were collected at 123 K and FOMBLIN was used as the cyroprotectant. Collected images were indexed and integrated using the HKL 2000 software^54^. *Sm*SDR crystals were characterized as belonging to the space P4_1_2_1_2, with the unit cell of *a* = *b* = 83.87, and *c* = 115.05 Å; α = β = *γ = *90°. The *Sm*SDR structures were solved by the molecular replacement methods by *AMoRe* module of CCP4 software^55,56^ using the *Sinorhizobium meliloti 1021* 3-oxoacyl-[acyl-carrier-protein] reductase structure (PDB code: 3V2G) as the search template. Rotation and translation functions followed by the rigid body refinement procedure were carried out using data from 8- to 3-Å. Crystallographic refinement was carried out using the *REFMAC5* module^57^ and coupled to *ARP/wARP*^58^ to add solvent molecules. 2*Fo–Fc* and *Fo–Fc* maps were produced and inspected after refinement and revised the model manually with the program ‘Coot’^59^. The overall quality of the final model was assessed by the program PROCHECK^60^. Table 2 shows the crystallographic statistics.

**Table 2:** Crystallographic statistics of *Sm*SDR variants.

	*Sm*SDR	F98AF202L	F98LF202L·NADPH	F98YF202Y
Beamline	NSRRC 15A1			
Wavelength (Å)	1.000			
Cell parameters				
*a*, *b*, *c* (Å)	83.87, 83.87, 115.05	83.80, 83.80, 114.25	84.01, 84.01, 115.82	83.93, 83.93, 115.04
α, β, *γ* (°)	90.00, 90.00, 90.00	90.00, 90.00, 90.00	90.00, 90.00, 90.00	90.00, 90.00, 90.00
Space group	P4_1_2_1_2	P4_1_2_1_2	P4_1_2_1_2	P4_1_2_1_2
Resolution (Å)	20.00–1.47	67.58–1.87	50.00–1.90	67.55–1.47
Completeness (%)^a^	99.8 (100.0)	99.5 (96.9)	92.3 (70.2)	98.8 (100.0)
Number of unique reflections	69956	34418	33651	70468
Redundancy^a^	11.4 (11.3)	13.7 (13.2)	26.7 (19.0)	28.1 (27.7)
R_merge_ (%)^a,b^	7.5 (93.1)	5.1 (21.2)	6.5 (23.1)	4.9 (17.2)
R_rim_ (%)	2.3 (28.3)	1.4 (6.0)	1.3 (6.1)	1.0 (3.7)
Average I/σ (I)^a^	36.66 (4.44)	44.73 (14.1)	46.01 (10.5)	85.1 (20.8)
CC_1/2_	0.991	0.998	0.993	0.996
Wilson B factor (Å^2^)	13.1	18.3	18.2	13.2
Refinement				
Resolution range (Å)	19.89–1.47	67.58–1.87	50.00–1.90	67.55–1.47
Number of atoms	3616	3565	3646	3666
R_factor_ (%)^c^	16.6	15.0	17.2	17.5
R_free_ (%)^d^	19.1	20.3	21.2	20.7
Bond length (Å)^f^	0.035	0.022	0.020	0.028
Bond angle (°)^f^	2.919	2.029	1.990	2.540
Refined B-factor (Å^2^)	18.23	20.61	22.54	20.094
Ramachandran analysis (%)^g^				
Favored/Allowed/Outliers	99/1/0	98/2/0	98/2/0	97/3/0
PDB code	4ZGW	5WUL	5WUW	5WVA

^*a*^Values in parentheses refer to statistics in the highest-resolution shell.

^*b*^*R*_merge_ = Σ|*I*_obs_ − <*I>*|/Σ*I*_obs_.

^*c*^*R*_*factor*_ = Σ|*F*_obs_ − *F*^*c*^_alc_|/Σ*F*_obs_, where *F*_obs_ and *F*_calc_ are the observed and calculated structure factor amplitudes, respectively.

^*d*^*R*_free_ was computed using 5% of the data assigned randomly.

^*e*^r.m.s.d., root mean square deviation.

^f^Root mean square deviation.

^g^Estimated standard uncertainties based on maximum likelihood.

The coordinates and structure factors have been deposited in the Protein Data Bank under the accession numbers 4ZGW (*Sm*SDR), 5WUL (F98A·F202L·*Sm*SDR), 5WUW (F98L·F202L·NADPH·*Sm*SDR) and 5WVA (F98Y·F202Y·*Sm*SDR).

### Sequence and Structural Comparisons

*Sm*SDR structures were compared with all protein structures in the DALI server (http://ekhidna.biocenter.helsinki.fi/dali_server/start). The secondary protein elements alignment was performed by ESPript tool version 3.0^61^ to generate the figures of the secondary elements alignment profiles. Structural comparison between the *Sm*SDR and its homologous structures was carried out using the program Lsqkab module^62^ in CCP4 program to superimpose Cα atoms. Structural figures were prepared with the program *PyMOL* (www.pymol.org).

### Virtual Docking

The model containing NADPH and HPMAE was prepared using the *Sm*SDR structure as the template. The NADPH binding residues within the 3.8 Å of the NADPH of the F98L·F202L·NADPH·*Sm*SDR were chosen as the binding pocket for docking NADPH using the GOLD module of Discovery Studio 2016^63,64^. Parameter settings were used as same as the described previously^33^. Docking procedures were terminated while three best solutions were obtained within a root mean square (r.m.s.) tolerance of 1.5 Å. Top ranking of the docked pose of the NADPH were chosen for superposing the orientation of the liganded NADPH. The NADPH pose with the lowest r.m.s of the superposition was selected as the second template for the HPMAE docking. The docked method of HPMAE was performed as well as that of NADPH with modification. The binding site was defined within 5 Å radius around the hydroxyl O atom the Tyr158 residue. Crucial residues for contacting the HPMAE are added to the criteria of the protein H-bond constraints. Top ranking of the docked pose of the HPMAE with the lowest energies was chosen for analysis.

### Enzyme activity assay

1-(3-hydroxylphenyl)-2-(methylamino) ethanone (HPMAE) was gifted from Industrial technology Research Institute, Hsinchu, Taiwan. The *Sm*SDR enzyme activity was measured by determining the consumption of NADPH using a 0.2 ml reaction mixture containing 12 mM HPMAE, 5 µM proteins with 50 mM sodium phosphate (pH 7.0), 300 mM NaCl. Each reaction was initialed by adding 0.4 mM NADPH, and subsequently monitored the extinction of NADPH fluorescence (λ_ex_ 340 nm, λ_em_ 460 nm) by CLARIO star ELISA reader. The steady-state kinetics analysis of *Sm*SDR was performed at 30 °C with the reaction mixtures as described above. The *K*_*m*_ and *k*_*cat*_ values were determined by plotting the initial velocity as a function of substrate concentration and fitting the plots to the Michaelis-Menten equation.

### Conversion of HPMAE to (*R*)-PE by *E*. *coli BL21* (*DE3*)

Conversion of HPMAE to (*R*)-PE was calculated according to the previous method^21^. The cell pellet was washed with 10% glycerol and resuspended in 10 ml of reaction mixture containing 5% (wet weight) cells, 10 mM HPMAE, 100 mM sodium phosphate buffer (pH 7.0) and 2% fructose. The bioconversion reaction of the whole cell was performed in a shaking flask at 30 °C to convert HPMAE to (*R*)-PE in one hours. The cells were then removed by centrifugation, and the supernatant was subjected to HPLC for the analysis of substrate consumption and product formation.

### Thermal shift assay

Thermal shift assays were performed according to Huynh and Partch^36^. The *Sm*SDR enzyme (5 μM) was incubated with 1000× dilution of SYPRO Orange in 50 mM sodium phosphate (pH 7.0) solution containing 300 mM NaCl. The mixture was transferred to a 96-well plate and spectra were collected at 0.3 °C intervals per minute from 25 °C to 95 °C on the Step One Plus Real-Time PCR System (Applied Biosystems™). The acquisition results were analyzed using GraphPad Prism. A melting temperature (Tm) is calculated from the denaturing curve.

### Ethical approval

This article does not contain any studies with human participants or animals performed by any of the authors.

## Electronic supplementary material

Supplementary Information
