# Supplementary material for: Structure-guided design of Serratia marcescens short-chain dehydrogenase/reductase for stereoselective synthesis of (R)-phenylephrine
Source: Sci Rep. 2018 Feb 2;8:2316. doi: 10.1038/s41598-018-19235-y (PMC5797133; doi:10.1038/s41598-018-19235-y)
Supplement: Supplementary file 1 — Supplementary Information [file 41598_2018_19235_MOESM1_ESM.pdf]

Supplementary Material for:

SREP-17-37597A

Structure-guided design of *Serratia marcescens* short-chain dehydrogenase/reductase for stereoselective synthesis of (*R*)-phenylephrine

Jai-Shin Liu[1], Yi-Chia Kuan[1,2], Yu Tsou[1], Tung-Yueh Lin[1],Wen-Hwei Hsu[2], Ming-Te Yang[2], Jong-Yih Lin[3], and Wen-Ching Wang[1*]

[1]Institute of Molecular and Cellular Biology & Department of Life Science, National Tsing Hua University, Hsinchu 300, Taiwan

[2]Institute of Molecular Biology, National Chung Hsing University, Taichung 402, Taiwan

[3]Deaprtment of Mechanical Engineering, National Chung Hsing University, Taichung 402, Taiwan

[*]To whom correspondence may be addressed: Wen-Ching Wang

E-mail address: wcwang@life.nthu.edu.tw; Tel: +886-3-5742766., Fax: +886-3-5715934.

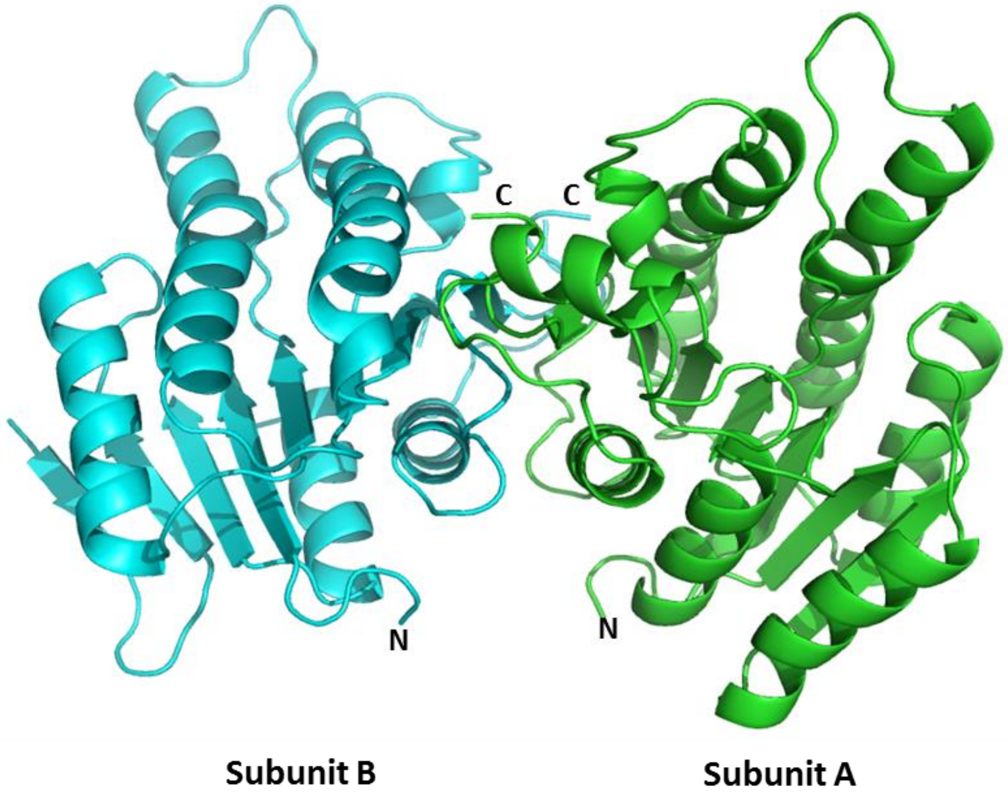

Fig. S1. Crystal structure of the apo-form *Sm*SDR consists of two subunits depicted in green and cyan, respectively. The N- and C-terminus are labeled N and C, respectively.

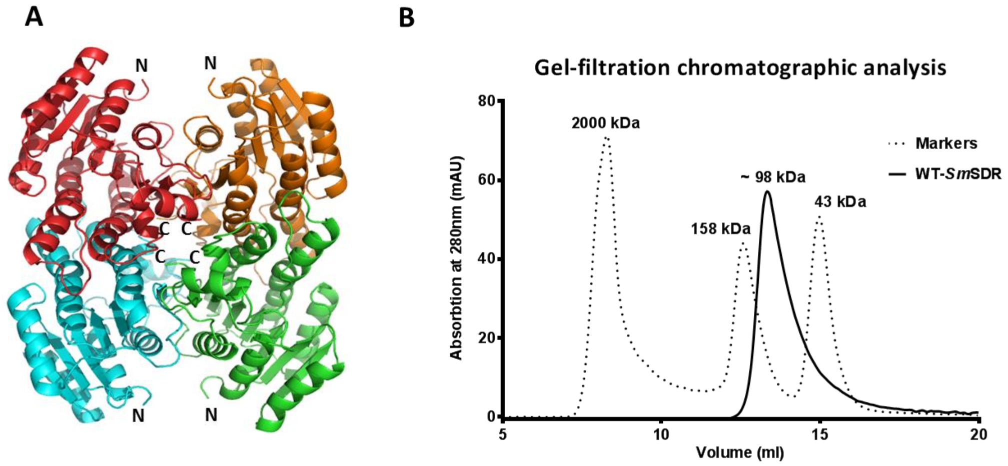

Fig. S2. (A) Tight tetramer formation in solution. (B) Gel-filtration chromatographic analysis of *Sm*SDR. The migrations of protein markers of known molecular weight (in kDas) are shown at the top of each peak shown in the chromatogram.

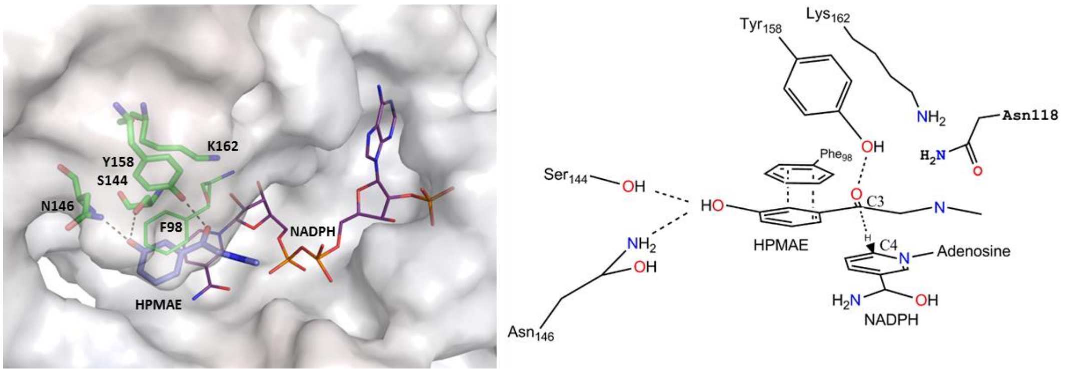

Fig. S3. Docking results for liganded HPMAE-*Sm*SDR protein. The HPMAE was docked into the binding site of NADPH-liganded *Sm*SDR protein by GOLD module of Discovery Studio 2016. The pose with the smallest r. m. s. was selected as final solution. The nearby residues (green) and NADPH (magenta) are shown as stick and the nitrogen, oxygen and phosphorus atoms are colored as blue, red, and orange, respectively. The HPMAE is depicted as purple stick model. Residues contacting with HPMAE are shown as thick sticks. Hydrogen bond interactions are shown by dash line with distance.

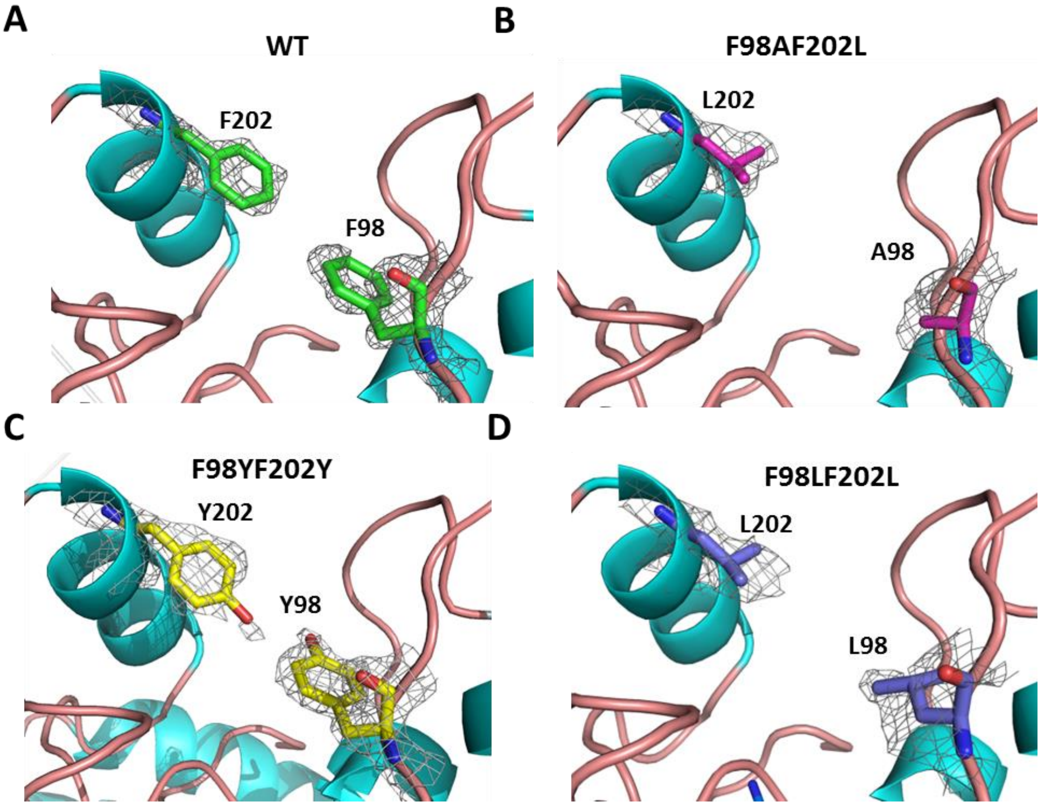

Fig. S4. Close-up view of the active site of the *Sm*SDR variants. (A) apo form *Sm*SDR. The double phenylalanine 98 and 202 are revealed as green sticks. (B) F98AF202L-*Sm*SDR. The replaced residues (Ala98 and Leu202) are presented as magenta stick models. (C) F98YF202Y-*Sm*SDR. The substituted Lue98 and Leu202 residues were are colored as yellow. (D) F98LF202L·NADPH-*Sm*SDR. The mutated Tyr98 and Tyr202 residues are illustrated as yellow stick-model. The α-helix and loop are colored as cyan and brown, respectively. The omit map that reveals density of residues 98 and 202 was contoured to 0.7 for wild-type and 1.0 σ for F98AF202L, F98LF202L, and F98YF202Y.

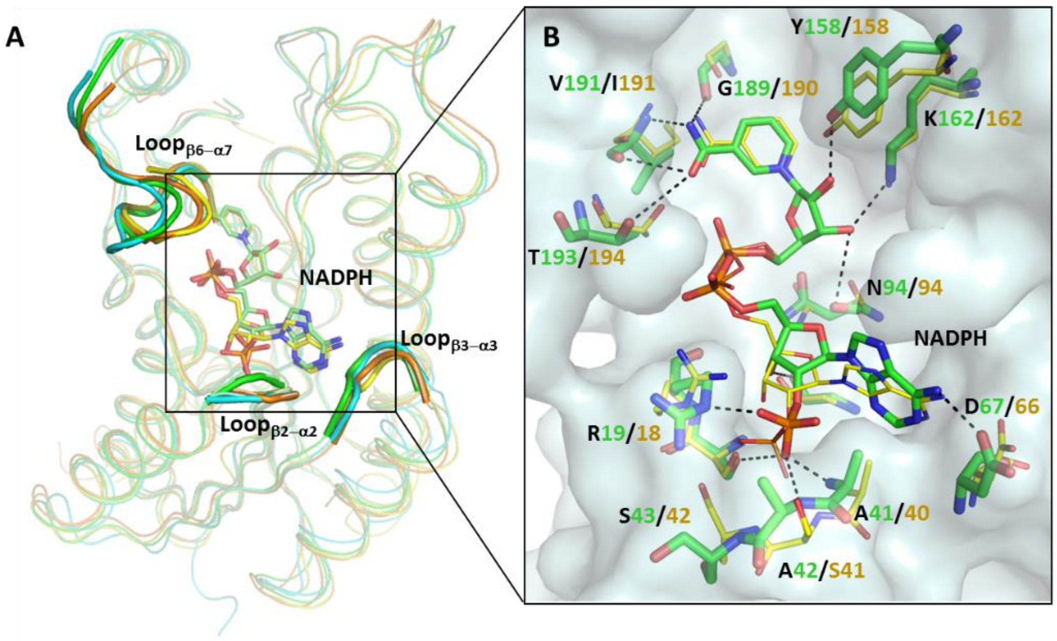

Fig. S5. (A) Superposition of *Sm*SDR (green) and *Se*OACPR (yellow). (B) Close-up view of the nucleotide binding sites between *Sm*SDR and *Se*OACPR. NADPH and interacting residues in *Sm*SDR are shown as heavy sticks, whereas the corresponding superimposed partners are shown thin sticks. The nitrogen, oxygen, and phosphate groups are colored blue, red, and orange, respectively. Hydrogen bonding is shown by dashed line.

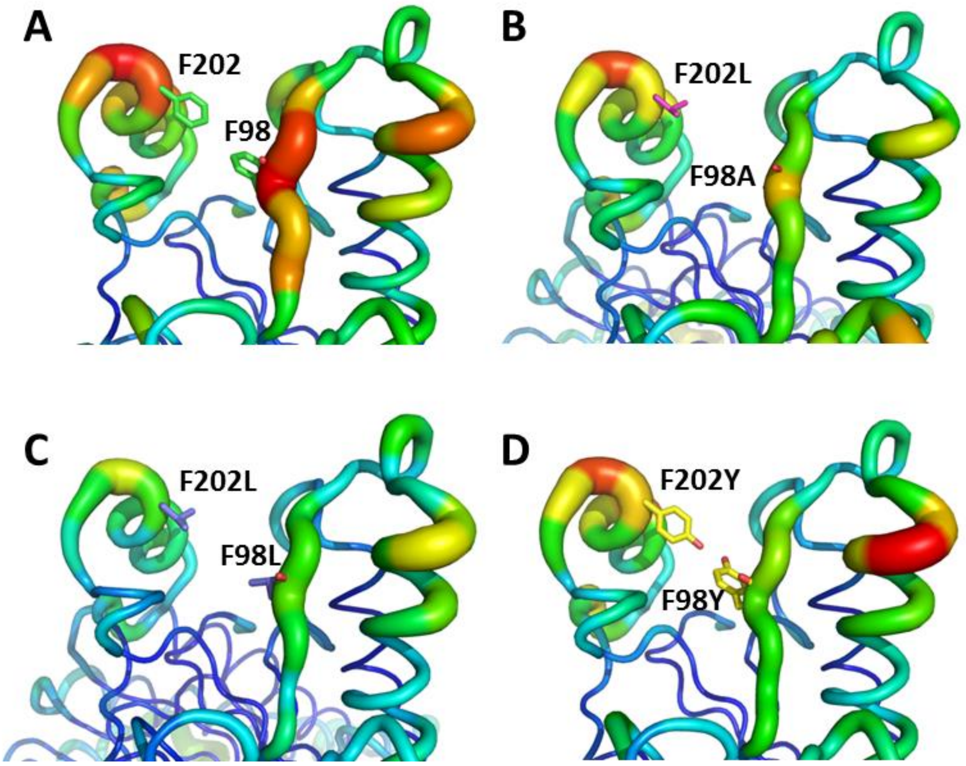

Fig. S6. Representation of structural mobility of in the two arms. (A) Apo form *Sm*SDR. (B) F98AF202L·*Sm*SDR. (C) F98LF202L·*Sm*SDR. (D) F98YF202Y·*Sm*SDR. Color orange to red and a wider tube indicate regions with higher values of B-factor, whereas blue and a thinner tube indicate regions with lower B-factor values.

Table S1. Oligonucleotide primers used in this study.

Primer for PCR	Sequence
*Sm*sdr-F[a]	5'- ATA*GGTACC*ATGACCACAGCACATCCTCTG -3'
*Sm*sdr-R[b]	5'- CGG*GTCGAC*CGCCGAGAAACCGCCGT -3'
Site-directed mutagenesis [c]	
*Sm*sdr-A42S-F	5'-ACGCCTCCTCGGCCGATCGCGCCG-3'
*Sm*sdr-A42S-R	5' CGGCGCGATCGGCCGAGGAGGCGT -3'
*Sm*sdr-F98A-F	5'- GAACAACGCCGGGGTAGCCACGCTGGGCGGC -3'
*Sm*sdr-F98A-R	5'- GCCGCCCAGCGTGGCTACCCCGGCGTTGTTC -3'
*Sm*sdr-F98L-F	5'- GAACAACGCCGGGGTACTTACGCTGGGCGGC -3'
*Sm*sdr-F98L-R	5'- GCCGCCCAGCGTAAGTACCCCGGCGTTGTTC -3'
*Sm*sdr-F98Y-F	5'- GAACAACGCCGGGGTATATACGCTGGGCGGC -3'
*Sm*sdr-F98Y-R	5'- GCCGCCCAGCGTATATACCCCGGCGTTGTTC -3'
*Sm*sdr-F202L-F	5'- GAACCCGGACGCCGGCGAGCTCGCCGATC -3'
*Sm*sdr-F202L-R	5'- GATCGGCGAGCTCGCCGGCGTCCGGGTTC -3'
*Sm*sdr-F202Y-F	5'- GAACCCGGACGCCGGCGAGTACGCCGATC -3'
*Sm*sdr-F202Y-R	5'- GATCGGCGTACTCGCCGGCGTCCGGGTTC -3'

[a] *Kpn*I site is indicate underlined.
[b] *Sal*I site is indicate underlined.
[c] The nucleotides of the mutated site are underlined.
